# NC CAN COVID-19 Action Network: Project ECHO in North Carolina

**DOI:** 10.1093/geroni/igab046.1896

**Published:** 2021-12-17

**Authors:** Ben Blomberg, Cristine Henage, Jennifer Hubbard, J Marvin McBride

**Affiliations:** 1 UNC Chapel Hill School of Medicine, Chapel Hill, North Carolina, United States; 2 The University of North Carolina at Chapel Hill, UNC, North Carolina, United States

## Abstract

Experts in geriatrics, infection control and nursing home administration joined the ECHO Hub team led by The Carolina Geriatrics Workforce Enhancement Program (CGWEP) at the University of North Carolina at Chapel Hill (UNC). Ninety-two of North Carolina’s 423 nursing homes enrolled in a 16-week videoconference series designed to address clinical, logistical, and leadership issues related to COVID-19. The CGWEP coordinated recruitment with two other Training Centers at UNC Family Medicine and the Mountain Area Health Education Center, reaching 58% of all NC nursing homes (N=245). Faculty used curriculum and pre-recorded videos provided by the Institute for Healthcare Improvement (IHI). Discussions demonstrated real-world problem solving as participants applied what they learned to local conditions. Quality Improvement (QI) experts from IHI mentored participants in gathering data and completing Plan, Do, Study, Act cycles to better respond to the challenges of COVID-19 among a critically vulnerable population.

